# Additively Manufactured Self-Healing Structures with Embedded Healing Agent Reservoirs

**DOI:** 10.1038/s41598-019-43883-3

**Published:** 2019-05-16

**Authors:** Keivan Davami, Mehrdad Mohsenizadeh, Morgan Mitcham, Praveen Damasus, Quintin Williams, Michael Munther

**Affiliations:** Department of Mechanical Engineering, Lamar University, Beaumont, Texas 77706 USA

**Keywords:** Mechanical engineering, Mechanical properties

## Abstract

Self-healing materials with the ability to partially or completely restore their mechanical properties by healing the damage inflicted on them have great potential for applications where there is no or only limited access available to conduct a repair. Here, we demonstrate a bio-inspired new design for self-healing materials, where unit cells embedded in the structure are filled with a UV-curable resin and act as reservoirs for the self-healing agent. This design makes the repeated healing of mechanical damage possible. When a crack propagates and reaches one of these embedded reservoirs, the healing agent is released into the crack plane through the capillary action, and after polymerization through UV light exposure, bonds the crack faces. The structures here were fabricated using a stereolithography technique by a layer-by-layer deposition of the material. “Resin trapping” as a unique integration technique is developed for the first time to expand the capability of additive manufacturing technique for creating components with broader functionalities. The self-healing materials were manufactured in one step without any needs for any sequential stages, i.e. filling the reservoir with the healing agent, in contrast with the previously reported self-healing materials. Multiscale mechanical tests such as nanoindentation and three-point bending confirm the efficiency of our method.

## Introduction

Drawing inspiration from nature for the purpose of solving complex engineering problems has been a technique employed by scientists and engineers for many decades. Many biological materials are hierarchical structures with sophisticated architectures, capable of self-healing and regenerating their functionality after the infliction of damage by external mechanical loads. While studies of biological systems continue to suggest new opportunities, fabrication challenges remain. However, research into applications of their analogs in real devices is becoming more prominent thanks to new developments in advanced manufacturing techniques. For instance, microvascular networks with complex and delicate architectures such as leaf venation^1–5^ and blood vascularization^6–9^ that are widely observed in biological systems for targeted delivery of nutrients for growth and healing, may also be replicated in transformative synthetic materials via various techniques, including soft lithography^10–12^, laser ablation^13,14^, and direct-write assembly^15^, to enable repetitive healing of damage.

Similar to the way that human skin undergoes repeated healing of isolated damage, self-healing materials are an artificially-synthesized class of materials capable of repairing damage once or even multiple times, countering degradation of the material and expanding its lifetime, reliability, and efficiency. They are of interest particularly for applications where long-term reliability is required in poorly accessible areas or the structure is subjected to repeated damage. Although most reports of self-healing materials are on polymers or elastomers, self-healing refers to any materials where mechanical damages are healed automatically or with minimum external intervention, resulting in full or partial restoration of the material’s mechanical properties^16^. This definition covers all classes of materials, including metals, ceramics, and cementitious materials that take advantage of the same underlying principles. It needs to be noted that after the material is amended, it is the functionality that is restored rather than the exact external shape or internal micro-structure^16^.

White *et al*.^17^ introduced self-healing polymer composites that incorporate microencapsulated healing agents along with an embedded catalyst. Upon introducing a microcrack to the microcapsule, the healing agent is released to fill the crack plane via capillary action. Healing was achieved when the healing agent is polymerized in contact with the catalyst. While including unterminated chain-end polymerization catalyst enables multiple healing events, when the capsules are depleted from healing agent, no subsequent healing was possible if a new crack were to occur. Also, the capsules need to have the right thickness since capsules with thin walls break while processing and capsules with thick walls do not break as the crack propagates. Toohey *et al*.^18^ fabricated a polymer-based structure with an embedded 3D microvascular network using a direct-wire assembly of a fugitive ink. The network was filled with a healing agent and upon crack formation in the coating, the agent, dicyclopentadiene (DCPD), is supplied by the network to the crack planes. In their design, the presence of the microchannel weakens the mechanical strength of the structure. In addition, several fabrication stages are needed to finalize the network. Limitation in the amount of the self-healing agents, as well as the requirement for the injection of the self-healing agent in the network and effectively sealing it are other downsides of this design. While this design enables the healing process for up to seven repairs, the self-healing is limited for any subsequent damages due to the depletion of the healing agent. Even though some of these issues were addressed in other work by the same group^19^, major challenges, such as the need for the two parts of the healing agents to be mixed diffusively within the crack plane and complicated fabrication procedure, still remain. In addition, the difficulty associated with the injection of not just one, but a few components of the healing agent in the network in these structures is a challenge. This limitation makes the need for an integration of micro valves and pumps inevitable for an efficient system^18^.

It remains an opportunity to explore the self-healing process in different materials for various applications and novel technological implementations. For instance, a combined microcapsule–microvascular system was recently reported to amend multiscale damages from an impact puncture of vascularized polymeric sheets^20^. Kang *et al*.^21^ reported a new class of self-healing stretchable polymeric material, crosslinked through precisely designed multistrength hydrogen bonding interactions, for potential applications in electronic (e-) skin. Kim *et al*.^22^ reported thermoplastic polyurethane (TPU) for possible implementation in the wearable electronics industry. The polymer-based structure was able to reobtain more than 75% of the mechanical properties of the virgin sample within 2 hours after splitting and bringing the two pieces in contact. Moreover, significant research efforts have been invested in self-healing cementitious materials^23–25^, composite materials^26–28^, and metals^29^.

Additive-manufacturing (AM) and the complexities enabled by it are revolutionizing manufacturing, further than just the geometry of the part, but also the chemistry and microstructure within the part with site-specific properties. Many polymer-based structures that are designed and fabricated by AM, however, tend to undergo damage that manifest commonly in the form of cracks, compromising the integrity of the structure and eventually limited functionality. The formation of these cracks, particularly deep within the structure, makes certification and qualification of additively manufactured polymer-based structures challenging, specifically since their detection and repair procedure are excessively prolonged and costly, if not impossible. Thus, while the AM technique is promising for various purposes such as low production-quantity parts with complex geometries, weight reduction of a system through part consolidation or topology optimization, design customization, rapid design iteration, product development, etc., the reliability issue remains to be addressed.

Herein, we introduce self-healing AM structures with remarkable mechanical performance and regenerative ability through the careful design and fabrication of the structure’s geometry. The structures reported here are capable of repair of sequential damages. The repair mechanism is triggered as soon as the crack propagates beneath the surface of the structure and reaches the reservoir of the healing agent. The self-healing process is based on the generation of a mobile phase healing agent, which is “trapped” during the fabrication in the reservoirs, thus no secondary stages are required. The healing agent leaks into the crack planes as a result of the capillary force, and the crack is closed upon exposure to the UV light, completing the healing process.

## Results and Discussion

**Figure 1:**
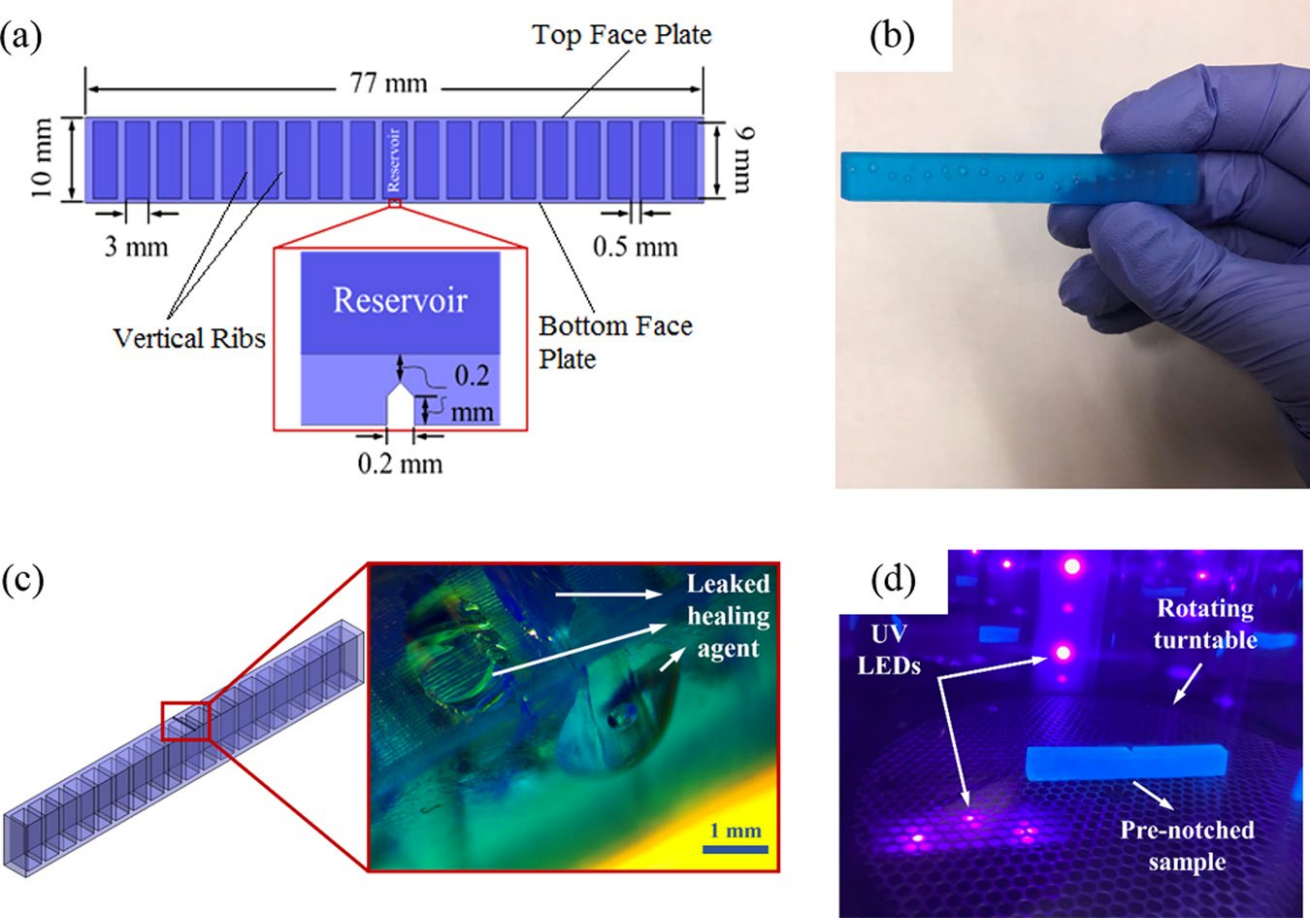
(**a**) A CAD model of the self-healing structure (front view). The inset shows the geometry of the straight notch, (**b**) an actual 3D printed specimen with hollow reservoirs and trapped resin, (**c**) a schematic of the structure with an optical image inset, showing the leaked healing agent from a crack, (**d**) curing the leaked UV-photocurable resin, using multi-directional UV LEDs.

To explore the novel biomimetic self-healing structures, a design that mimics the architecture of the one seen in puntia, also called prickly pear (a member of the cactus family^30,31^) was implemented and fabricated. The self-healing structures were produced from a UV-curable resin via a stereolithography (SLA) AM method (Section I and Movie S1, Supplementary Information,). The structures were designed with unit cells comprising vertical ribs (Fig. 1a). The unit cells act as reservoirs that contain the healing agent. Due to the layer-by-layer nature of this 3D printing method and the design of the reservoirs, some resin is “trapped” inside of each unit cell without becoming cured as illustrated in Fig. 1b (Movie S2, Supplementary Information). This uncured resin acts as a healing agent inside the self-healing structures, right beneath the top face plate. Similar to almost all opuntias with flattened pads (cladodes) that when cut, the mucilage cells ooze out, closing the opened area^30^, as soon as a crack forms and propagates to the reservoir of the self-healing agent, the agent wicks into the crack plane due to the capillary forces. The UV-photocurable resin has a viscosity of 850–1000 cps at 25 °C that promotes flow into the crack opening. This small amount of resin becomes cured upon being exposed to the UV light for a short amount of time (<120 sec) from a UV-light source. Figure 1c shows the healing agent leaking out of a crack and Fig. 1d depicts the curing procedure.

The short curing time and consequently quick healing process is advantageous for these self-healing structures with embedded healing agent reservoirs compared to many other previously reported self-healing materials. For instance, self-healing materials with microvascular networks reported by Toohey *et al*.^18^ needed to be kept at room temperature for a period of 12 hours in order to become healed. Self-healing materials with interpenetrating microvascular networks reported by Hansen *et al*.^32^ had to go under cyclic bending (50 cycles at 100 *μ*m displacement) to enhance the mixing of the healing agents at the location of the crack, and after that required to be subjected to 48 hours of curing at 30 °C.

Various mechanical tests were conducted to investigate the healing capability of the structures. For each test, three identical samples with an overall dimension of 5 × 10 × 77 mm (H × W × L) were printed using a top-down SLA-based 3D printer. A notch with equal width and depth of 200 µm was incorporated in the middle of the CAD (computer aided design) model of two out of three specimens (Fig. 1a). This notch enhances the repeatability of the experiments and encourages the initiation of a straight crack under flexural (3-point bending) tests^32^. When a crack forms, propagates, and reaches the reservoir, the resin wicks into the crack planes as a result of capillary forces and closes the crack when it becomes cured under exposure to UV light. These forces are not high enough to drain out and deplete the large amount of healing agent in the reservoirs. The agent’s relatively high viscosity, approximately 850–1000 cps at 25 °C, further aids in limiting its flow out of the damaged area. After healing, the specimen is tested again, and a new crack is formed under a new critical load and the aforementioned process is repeated. The small amount of leaked healing agent in the self-healing samples becomes cured relatively quickly under the UV radiation with a wavelength of 405 nm. A UV-light source was employed to cure the leaked-healing agent for 3 min. at 50 °C. To compare the effectiveness of the capillary forces for filling the crack, the notch of the second sample was manually filled before the tests. The last unnotched specimen (virgin) remained unfilled and was tested to provide a reference. It is worth noting that for simplicity’s sake, the structures were placed into a UV oven for curing; however, other types of UV sources can initiate and complete the healing process. In the case of a difficult to access part, on-site repair can be easily implemented using a remote UV source. Additionally, unloading the structure is not necessary to the healing process. A damaged structure is able to cure under loading as the healing mechanism is not affected.

**Figure 2:**
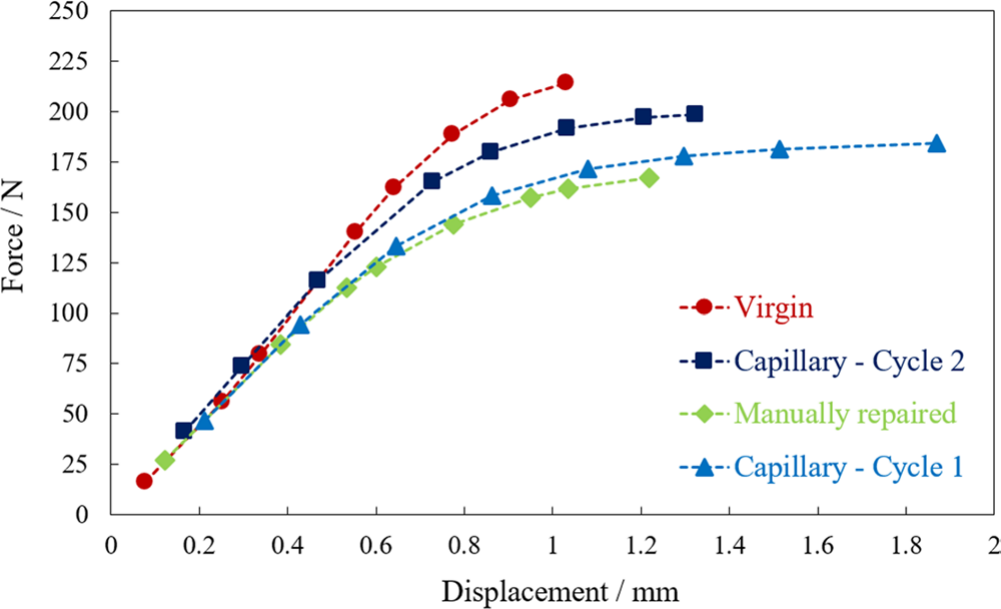
Force-displacement curves of three different types of specimens under the tensile loads.

The samples underwent tensile tests following the ASTM D638 standard and their force-displacement curves were recorded at a constant crosshead speed of 13 mm min^−1^. Figure 2 shows the force-displacement curves for each specimen type. There was a difference of 22% between the tensile fracture load of the virgin specimen without a notch and the sample that was manually repaired. By comparing the fracture force of the healed sample before and after healing (182 N for Capillary – Cycle 1 and 199 N for Capillary – Cycle 2), it can be seen that the fracture force increases by around 17 N after the sample was repaired. There is a significant difference between the fracture force of the manually repaired sample and the sample after healing (Capillary – Cycle 2). This indicates that the self-healing process is effective and is reviving the original mechanical performance of the structure.

**Figure 3:**
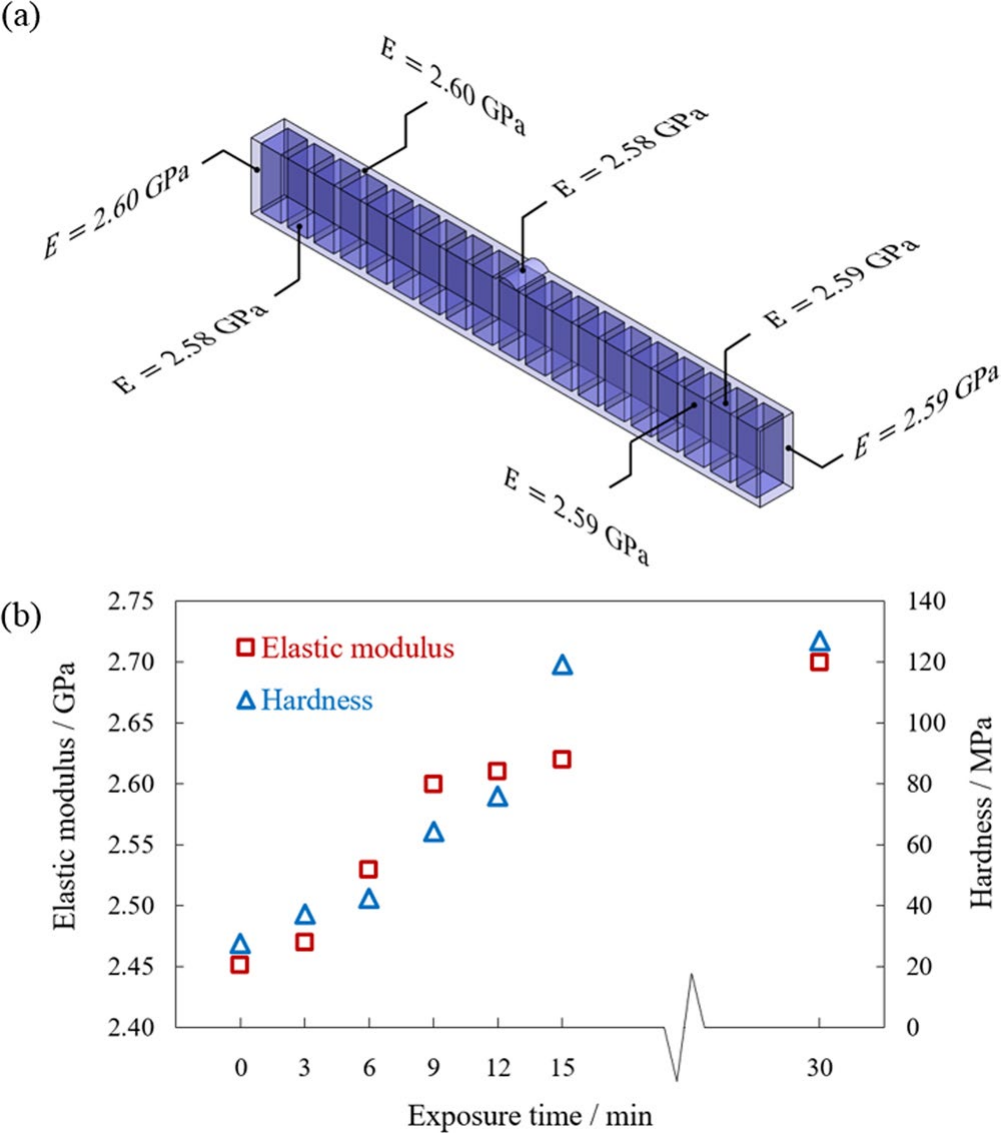
Nanoindentation test results. (**a**) The elastic modulus of different locations on a cured specimen after 9 min exposure to a 405 nm UV light, (**b**) elastic modulus and hardness versus the exposure time for a cured specimen under the UV light.

In order to evaluate how the properties of the healed location differs from the rest of the structure, the site-specific nanomechanical properties of the healed crack and its vicinity were evaluated and compared with the rest of the structure, using a nanoindentation technique (Method and Section II, Supplementary Information). As can be seen in Fig. 3a, the cured healing agent at the crack location has an elastic modulus in the range of that for the rest of the structure. The effect of the exposure time on the elastic modulus and hardness of the sample is shown in Fig. 3b. As can be seen here, with an increase in the exposure time, both the elastic modulus and hardness increase until the sample becomes fully cured.

**Figure 4:**
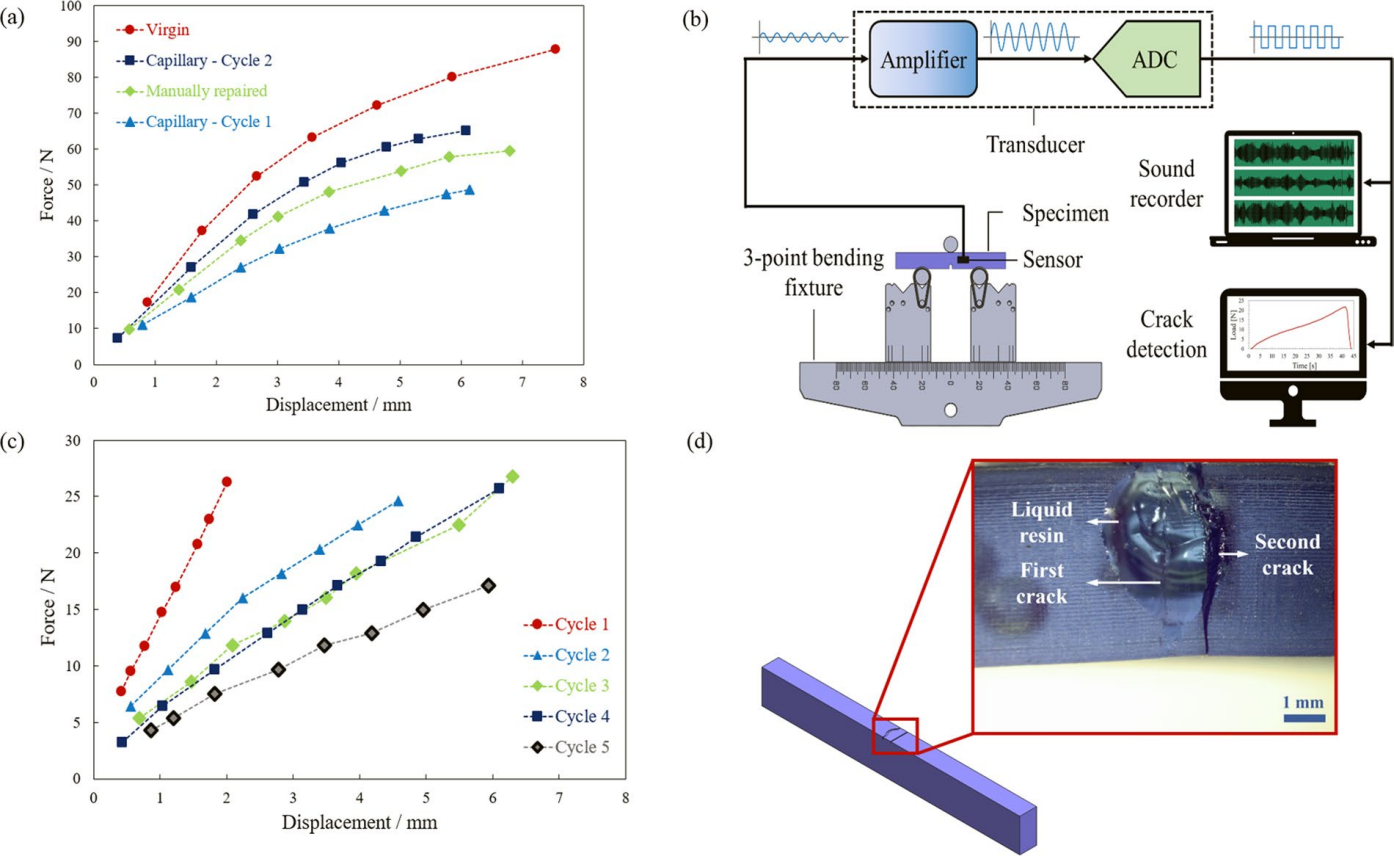
(**a**) Force-displacement curves from the 3-point bending of the specimens before and after healing process (Cycle 1, Cycle 2, respectively) as well as that for manually repaired and virgin samples, (**b**) a schematic of the experimental procedure for the crack detection using an acoustic sensor, (**c**) force-displacement curves for one specimen exhibiting four continuous healing cycles before fracture, (**d**) a schematic and an optical image of the self-healing structure after a crack is formed and the healing agent leaked out to the surface. The inset shows a zoomed-in OM image of the first and second crack.

Figure 4a compares 3-point bending force-displacement responses of the self-healing structure before (Capillary – Cycle 1) and after a crack was formed, healed, and cured (Capillary – Cycle 2) under the UV light, with those for virgin, and manually repaired specimens. Although incorporating a notch into the virgin specimen lowers its bending load capacity for about 44% (compare Virgin and Capillary – Cycle 1), the notched specimen can be strengthened through either a manually self-repair or self-healing mechanism, for up to 22% and 32%, respectively. Again, the higher increase in the fracture load for self-repaired samples in comparison with that for the manually repaired ones under 3-point bending indicates the efficiency of our method.

One of the main issues of self-healing materials is their limited number of self-repairs, commonly attributed to the depletion of the healing agent in the vicinity of the crack^18,32^. To assess the healing capability of the structure under sequential damages, cyclic 3-point bending tests were conducted on a notched specimen at a monotonic crosshead speed of 13 mm min^−1^, following the ASTM D5045 standard. In order to accurately detect the occurrence of the crack, a high-impedance piezo electric transducer (AD-35 by Adeline) along with a recording interface (iRig Pre) were used (Fig. 4b). The transducer senses audio vibrations through direct contact with the specimen under the test as soon as the crack initiates, while filtering the air vibrations (Figure S5, Section III, Supplementary Information). An audio recording program was used to record the output for further analysis and the detection of the crack initiation time. A correlation was made between the collected data from the sensor and the recorded force-time data (Figure S6, Section III, Supplementary Information). Upon the initiation of the crack, the load at which the crack was reopened was recorded and the specimen was exposed to the UV light and cured for 3 min at 50 °C. By doing this, the crack was successfully healed, and the test was repeated for further cycles, under the same testing conditions, until the sample fractured completely.

As can be seen in Fig. 4c, with more cycles, the sample’s stiffness degrades and the crack initiates at a smaller load since with additional loadings microcracks form and propagate through the specimen, while might not even reach the reservoirs. These microcracks affect the integrity and as a result the strength of the structure. The fracture location was carefully evaluated in self-healed specimens. It was noticed that the self-healing structures failed at a new location rather than where the initial repair was conducted (Fig. 4d), indicating that the specimens, were able to restore their homogeneity after the repair. It is worth noting that the ability for the structure to heal multiple times is attributed to the availability of a large amount of healing agent. Two factors control the flow of the agent from the damaged region. The material’s relatively high viscosity of approximately 850–1000 cps at 25 °C aids in introducing only a small amount of agent into the crack. Additionally, the curing of the agent upon exposure to UV light also slows the flow of the healing material, ultimately halting it once fully cured. While theoretically the healing can continue as long as there is healing agent left in the reservoirs, the structure might fail due to a catastrophic fatigue fracture.

**Figure 5:**
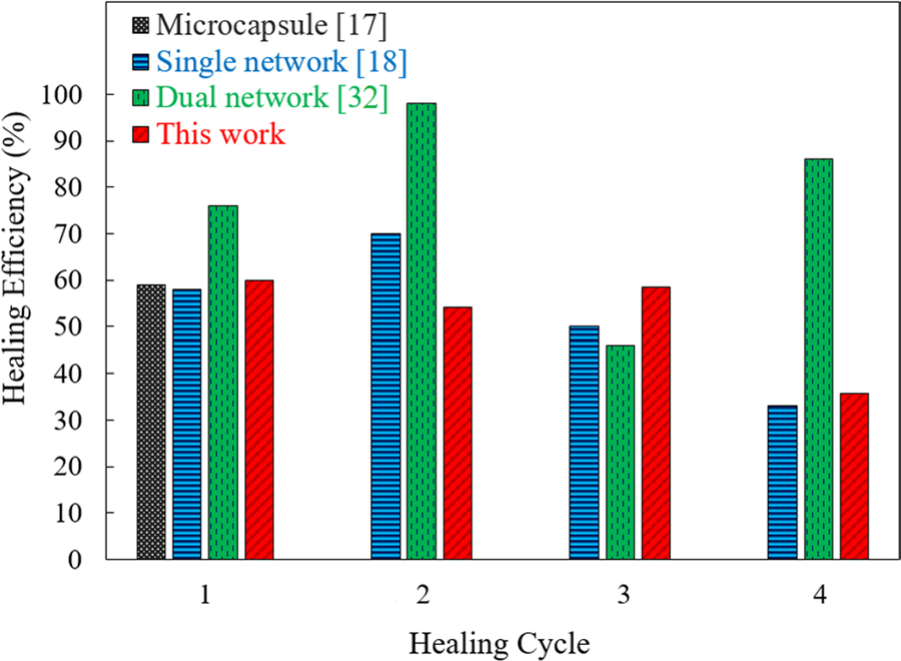
The healing efficiency of the specimens for each cycle of loading, compared with reported data from microcapsule (black)^17^, single network (blue)^18^, and dual network (green)^32^.

The healing efficiency, η, is commonly stated as the ratio of fracture toughness of the material after healing to the original fracture toughness. In this work, the healing efficiency was calculated from the ratio of the fracture force of the structure after healing to the fracture force of the as-printed structure. A similar approach was implemented in previous reports^18,32^. The time at which the crack initiated was used and the corresponding load was recorded to calculate the healing efficiency for each loading cycle. The healing efficiency for the first cycle was measured to be approximately 60% and an average healing efficiency of 52% was obtained for all four cycles. Figure 5 compares the healing efficiency of the structures reported here with the ones reported before. Our results surpass the ones reported previously for self-healing structures with microcapsules^17^ as well as those for an interpenetrating single-network microvascular system^18^. The healing efficiency of our design is less than that of dual-network microvascular systems^32^, however, significantly shorter preparation and healing time make the design reported here excel when compared to other reported self-healing structures.

## Conclusion

In summary, we demonstrated a structure capable of self-healing thanks to its biomimetic architecture. These novel structures were created through a stereolithography AM technique as it facilitates the fabrication of complex structures. Healing is accomplished by incorporating reservoirs of UV-curable resin that act as a self-healing agent within the structure. The reservoirs are able to provide the healing agent that is trapped inside them during the fabrication for multiple damage sites or sequential damages. The damage-induced triggering mechanism offers site-specific healing. When a crack forms, the healing agent leaks out to the crack location due to the capillary force. The small amount of UV-curable resin becomes cured when is exposed to the UV light for a short period of time. After a sufficient exposure time, the cracks are repaired, and the structural integrity of the specimen is restored. As the crack reopens under subsequent loading or new cracks form, the healing cycle is repeated. The average healing efficiency of the structures introduced here was ~52%. This value compares favorably with that for the previously reported self-healing materials. Self-healing of the microcracks not only alleviate the effects of environmentally- and functionally-promoted crack initiation such as the stress corrosion cracking, thermal expansion cracking, and fatigue-related cracking, but also mitigate the need for inspection methods. The design reported here can enhance the reliability and working life of many polymer-based additively-manufactured materials. This platform enables not only new avenues for continuous delivery of healing agents for self-repair of a damage that can occur multiple times, but also offers a new way of embedding other active species for additional functionalities.

## Methods

### Fabrication process

Test specimens (Figure S1) were fabricated using a Form2 3D printing system (Formlabs Inc., USA) top-down stereolithography technique. The fabrication started with the design of the specimen in SolidWorks. Stereolithography (STL) files were generated and transferred to the printer’s software (PreForm). The STL file is virtually sliced into several interconnected 2D layers, with a layer thickness (resolution) spanning from 25 to 200 *μ*m. The processed data is then uploaded to the printer to start the layer-by-layer additive-manufacturing process, through spatially-controlled polymerization of an ABS-like UV-curable photopolymer resin (commercially named as Tough resin). The mechanical properties of the polymer can be seen in Table S1. To ensure a successful print, the liquid resin is preheated and maintained at a constant temperature, before and during printings. The printing process begins with trapping a thin-film of the liquid resin, between a build platform and the bottom of a resin tank. The thin-film is exposed to an ultraviolet (UV) laser beam with 405 nm wavelength which is guided in XY directions via a galvanometer system. Upon exposure, the liquid resin is solidified at predefined site-specific locations, based on the STL data supplied to the machine, to pattern the desired path on the build platform. The layer thickness of the product is automatically controlled by adjusting the UV exposure time of the resin. Before starting the next printing cycle, a sliding mechanism gently detaches the formed solid layer from the bottom of the tank, while the build platform that now has the printed layer attached to it, is raised to allow the resin to be stirred by a wiper to remove the bubbles. This photopolymerization process repeats until the entire 3D structure is fabricated. After the printing, the structures are agitated in an isopropyl alcohol (IPA) bath, to remove excess uncrosslinked resin.

### Macromechanical tests

The tensile tests were carried out using a Mark-10 ESM 303 Motorized Tension/Compression Test Stand, equipped with a 1 kN force gauge (Model M4-200) and 5.3 kN grips. The specimens were subjected to quasi-static uniaxial tensile loads, at a constant crosshead speed of 13 mm min^−1^. Additionally, the samples underwent 3-point bending tests according to ASTM D5045 standard. Results were recorded using a built-in MESUR®gauge Plus software.

### Nanomechanical characterization

Nanomechanical experiments were conducted using a Hysitron TI 980 Triboindenter (Hysitron, Minneapolis, USA), equipped with a 3D OmniProbe/MultiRange NanoProbe (MRNP) high-load transducer, at room temperature. A high-load Berkovich diamond tip with a curvature radius of 150 nm, the total included angle of 142.35° and the half angle of 65.35°, was used for the characterization. The indenter was calibrated using a standard polycarbonate sample. A series of indentations was conducted using a standard, quasi-static trapezoidal loading function (Figure S2). For each indentation, the trapezoidal loading function increased the load across a 30 second time period to a peak load of 20 mN, and then held that peak load constant for a period of 15 seconds in order to reduce the effects of drift and creep of the material, allowing it enough time to settle, before unloading gradually in another 30 second period. In total, three measurements were conducted for each point and the average value was reported. An example loading curve is shown in Figure S2. A representative example of a single indent of this function on the self-healing structures is shown in Figure S3.

### Crack initiation detection

The specimens were tested in a 3-point bending setup to induce fracture in the structure at the location of the notch while the crack initiation was recorded by an acoustic emission sensor (Figure S5). The acoustic-emission sensor (AD-35 by Adeline) accompanied with a recording interface (iRig Pre) was implemented to detect the occurrence of crack events during the healed-specimen tests. Data from the acoustic-emission sensor was collected and exported to a computer to establish a correlation with the load-time data in order to determine the peak load at which the crack initiated. The sensor outputs information as ‘signal versus time,’ and in order to make a correlation between the sensor data and the F-D results, force-time was recorded. Figure S6 shows this correlation.

After thorough analysis of the sound files, the data concluded consistent wave forms where the crack initially took place after each test. Extraneous noise was able to be processed through a frequency filter, which removed noise from the tensile machine and enhanced frequencies from when the crack initiated. Frequencies from 140 Hz to 18000 Hz were removed and frequencies from 100 Hz to 139 Hz were boosted significantly in order to precisely detect the specimen crack. This filter and equalization method were applied to each additional recording of the sample. A parametric EQ and filter plug in were used in Logic Pro recording software program.

## Supplementary information

Movie S1

Movie S2

Supplementary Information

## Data Availability

All relevant data and code are available from the authors upon request.
