# Supplementary material for: Additively Manufactured Self-Healing Structures with Embedded Healing Agent Reservoirs
Source: Sci Rep. 2019 May 16;9:7474. doi: 10.1038/s41598-019-43883-3 (PMC6522478; doi:10.1038/s41598-019-43883-3)
Supplement: Supplementary file 3 — Supplementary Information [file 41598_2019_43883_MOESM3_ESM.docx]

**Supplementary Information:**

**Additively Manufactured Self-Healing Structures with Embedded Healing Agent Reservoirs**

Keivan Davami*, Mehrdad Mohsenizadeh, Morgan Mitcham, Praveen Damasus, Quintin Williams, Michael Munther

Department of Mechanical Engineering, Lamar University, Beaumont, Texas 77706, USA

* Corresponding Author: Keivan Davami ([kdavami@lamar.edu](mailto:kdavami@lamar.edu))

**Section I: Fabrication Process**

**Table S1.** Tough resin physical properties.

| **Mechanical Properties** | **Without**  **post cure** | **Post-cured** | **Method** |
| --- | --- | --- | --- |
| Ultimate Tensile Strength | 34.7 MPa | 55.7 MPa | ASTM D 638-14 |
| Tensile Modulus | 1.7 GPa | 2.7 GPa | ASTM D 638-14 |
| Elongation at Break | 42 % | 24 % | ASTM D 638-14 |
| Flexural Strength at 5% Strain | 20.8 MPa | 60.6 MPa | ASTM D 790-15 |
| Flexural Modulus | 0.6 GPa | 1.6 GPa | ASTM D 790-15 |
| Notched IZOD | 32.6 J/m | 38 J/m | ASTM D256-10 |

| 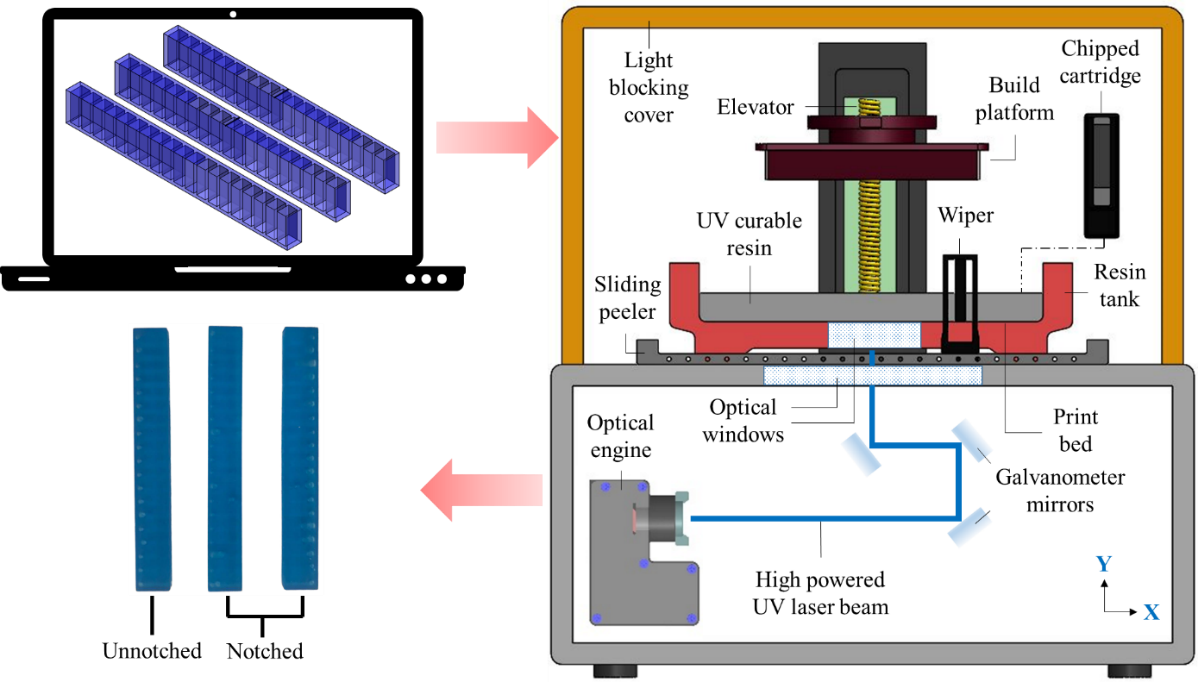* The data in this table has been provided by *Formlabs*. |
| --- |
| **Figure S1.** A schematic of the stereolithography (SLA) additive-manufacturing procedure. |

**Section II****:** **Nanomechanical Characterization**

| 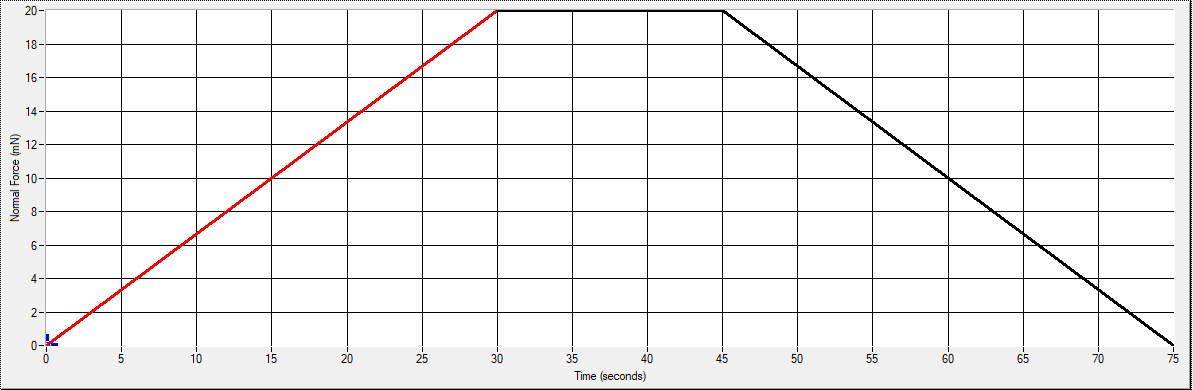 |
| --- |
| **Figure S2.** A screen capture showing the quasi-static trapezoidal loading function. |

| 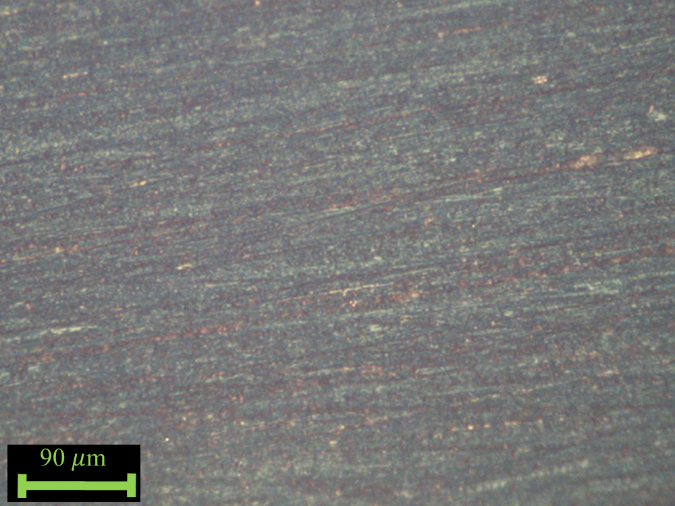 | 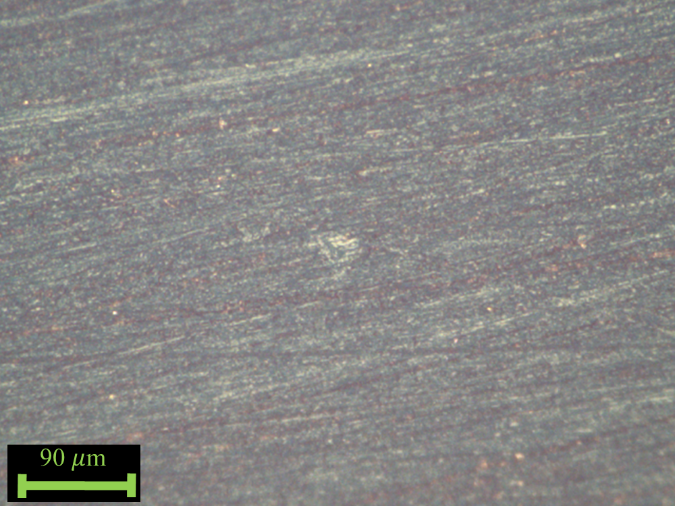 |
| --- | --- |
| **Figure S3.** The surface of the specimen before (a) and after (b) the nanoindentation test. The red circle encompasses the location of the indent. | |

Figure S4 shows a representative force-displacement curve. The reduced elastic modulus and hardness were calculated from the slope of the unloading section of the curve according to Oliver and Pharr methodology using a curve-fitting method. The curve fitting parameters were adjusted to different confidence intervals in order to more precisely obtain true values.

The Young’s modulus ($E_{s}$) of the material can be obtained from:

| $\frac{1}{E_{r}}=\frac{1-{\nu_{s}}^{2}}{E_{s}}+\frac{1-{\nu_{i}}^{2}}{E_{i}}$ | (1) |
| --- | --- |

Where $E_{s}$ is the Young’s modulus of the specimen, $E_{i}$ is Young’s modulus of the indenter, $\nu_{s}$ is the Poisson’s ratio of the specimen, and $\nu_{i}$ is the Poisson’s ratio of the indenter. For diamond Berkovich indenter tip, $E_{i}=1140$ GPa and $\nu_{i}=0.07$. $E_{r}$ is the reduced modulus given by indenter and $\nu_{s}=0.34$ of the sample.

| 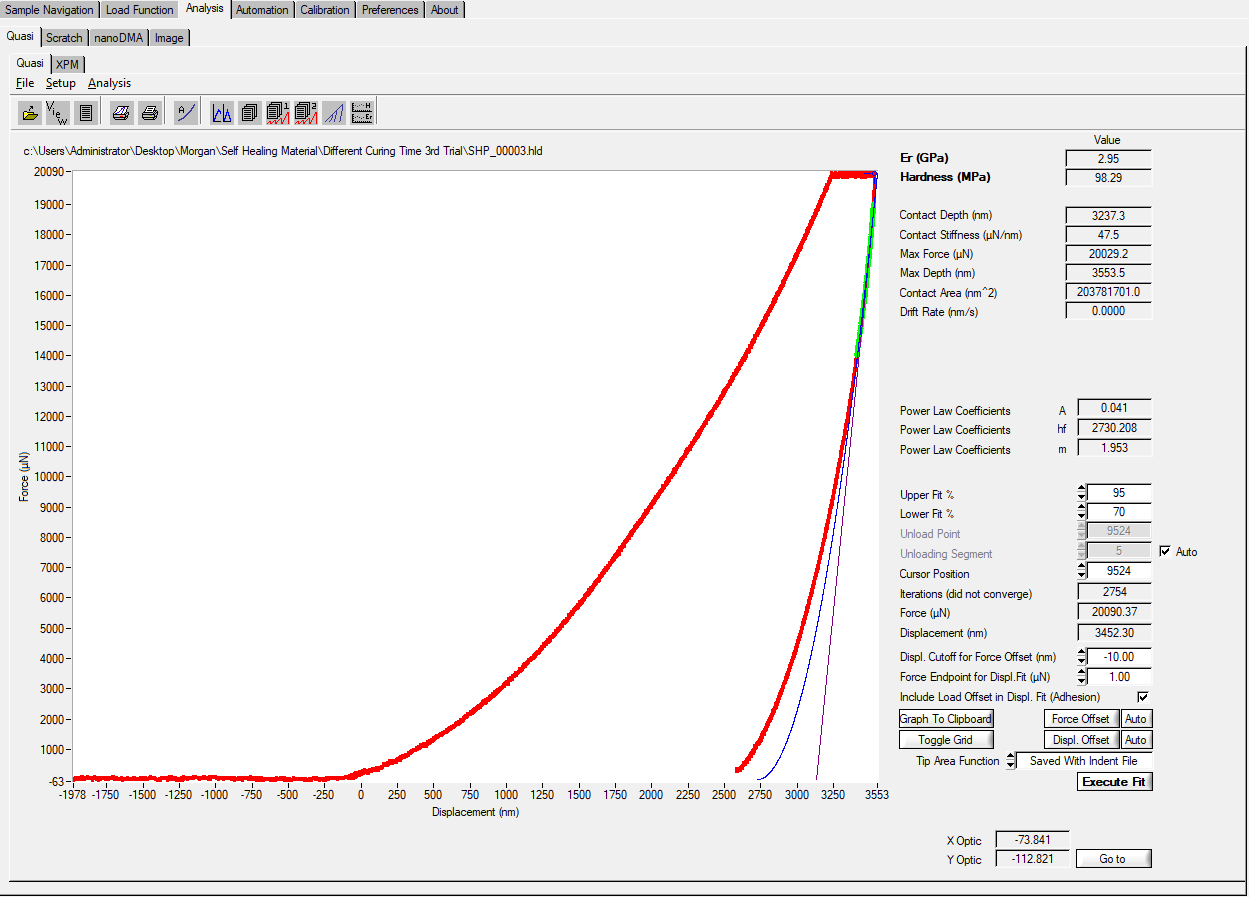 |
| --- |
| **Figure S4.** A screen capture of curve fitting on nanoindentation force-displacement curve, showing resulting elastic property values. |

**Section III:** **Crack Initiation Detection Setup**

**
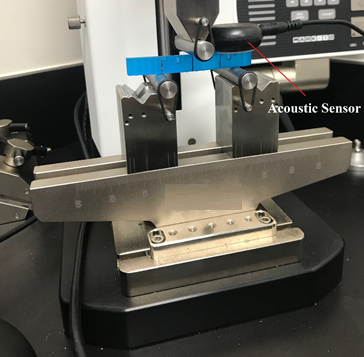
**

**Figure S5.** A self-healing specimen placed in a 3-point bend geometry with acoustic emission sensor.

**
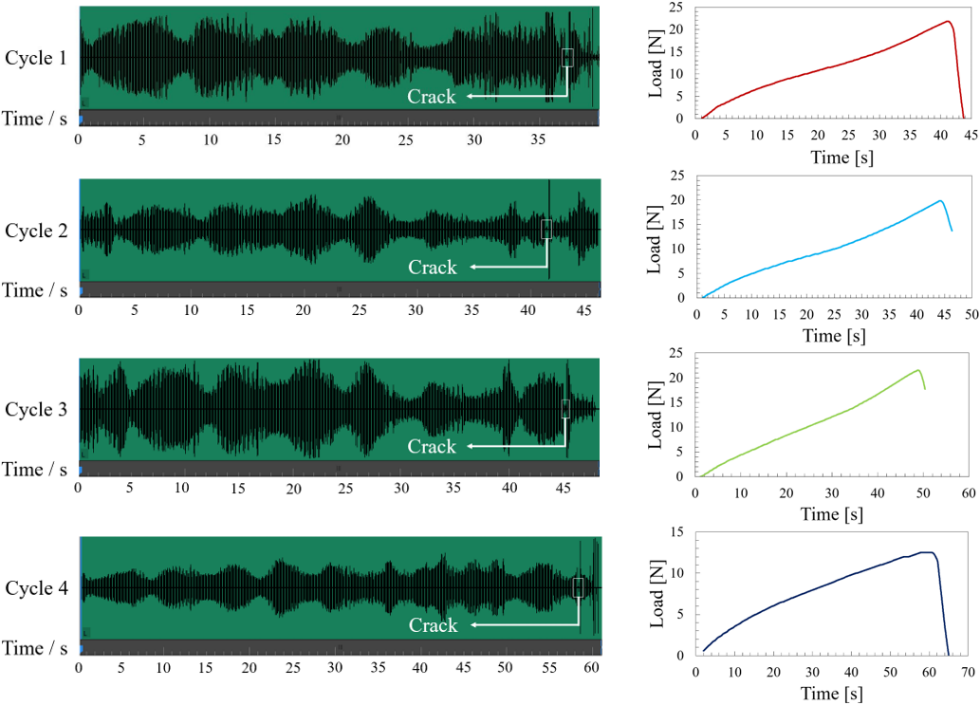
**

(a) (b)

**Figure S6**. (a) The output audio signals detected by the acoustic sensor, (b) corresponding force-time graphs.
